# Anti‐inflammatory hydrogel dressings and skin wound healing

**DOI:** 10.1002/ctm2.1094

**Published:** 2022-11-10

**Authors:** Can Huang, Lanlan Dong, Baohua Zhao, Yifei Lu, Shurun Huang, Zhiqiang Yuan, Gaoxing Luo, Yong Xu, Wei Qian

**Affiliations:** ^1^ Institute of Burn Research Southwest Hospital State Key Laboratory of Trauma Burn and Combined Injury Chongqing Key Laboratory for Disease Proteomics Army Medical University Chongqing China; ^2^ Department of Burns and Plastic Surgery the 910th Hospital of Joint Logistic Force of Chinese People's Liberation Army Quanzhou Fujian China; ^3^ Orthopedic Institute Suzhou Medical College Soochow University Suzhou China; ^4^ B CUBE Center for Molecular Bioengineering Technische Universität Dresden Dresden Germany

**Keywords:** anti‐inflammatory, chemokines, hydrogel dressings, reactive oxygen species (ROS), wound healing

## Abstract

Hydrogels are promising and widely utilized in the biomedical field. In recent years, the anti‐inflammatory function of hydrogel dressings has been significantly improved, addressing many clinical challenges presented in ongoing endeavours to promote wound healing. Wound healing is a cascaded and highly complex process, especially in chronic wounds, such as diabetic and severe burn wounds, in which adverse endogenous or exogenous factors can interfere with inflammatory regulation, leading to the disruption of the healing process. Although insufficient wound inflammation is uncommon, excessive inflammatory infiltration is an almost universal feature of chronic wounds, which impedes a histological repair of the wound in a predictable biological step and chronological order. Therefore, resolving excessive inflammation in wound healing is essential. In the past 5 years, extensive research has been conducted on hydrogel dressings to address excessive inflammation in wound healing, specifically by efficiently scavenging excessive free radicals, sequestering chemokines and promoting M_1_‐to‐M_2_ polarization of macrophages, thereby regulating inflammation and promoting wound healing. In this study, we introduced novel anti‐inflammatory hydrogel dressings and demonstrated innovative methods for their preparation and application to achieve enhanced healing. In addition, we summarize the most important properties required for wound healing and discuss our analysis of potential challenges yet to be addressed.

## Graphical abstract

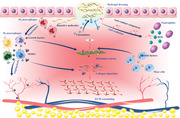

## 1 INTRODUCTION

As one of the most important organs of the human body, which protects us from harsh external environments, the skin is often damaged by traumas, severe burns, ulcers and various other injuries, thus disrupting its protective barrier functions and vital role in sensory perception. In addition, such injuries affect a patient's mental health and constitute an enormous societal economic burden.^1^, ^2^ Therefore, identifying effective therapeutic strategies to promote wound healing is an extremely urgent requirement.

### 1.1 Wound healing process

When skin is damaged and a wound is formed, the body initiates the healing process, consisting mainly of haemostasis, inflammation, proliferation and remodelling.^3^, ^4^ The haemostatic process involves some highly complex biological activities. First, the small blood vessels and capillaries around the wound reactively constrict to reduce local blood flow.^5^ Subsequently, the platelets are attracted to aggregate into blood clots by the exposed collagen fibres. Concomitantly, the platelets will release vasoactive substances, such as 5‐hydroxytryptamine and prostaglandins, which further constrict blood vessels and slow blood flow. Meanwhile, the phospholipids and adenosine diphosphate released by platelets will attract more platelets to aggregate into blood clots. Finally, the endogenous and exogenous coagulation processes are initiated.^6^, ^7^, ^8^

The followed stage is the inflammatory phase. The inflammatory response is characterized by increased vascular permeability and activated inflammatory cells, such as monocytes, lymphocytes and neutrophils, migrating to the wound in response to chemokines. The inflammatory mediators and inflammatory cells are essential for the removal of necrotic tissue and foreign bodies, and initiation and regulation of wound repair.^7^, ^8^, ^9^

The proliferative phase, closely linked to the inflammatory phase, is a prerequisite for re‐establishing skin barrier function.^9^, ^10^, ^11^ The main cells, involved in the process of skin reconstruction, are keratinocytes, fibroblasts and vascular endothelial cells. These cells accomplish wound epithelial regeneration, neovascularization and granulation tissue formation through their proliferation and migration activity.^12^

The remodelling phase, involving the maturation and reconstruction of nascent tissues, is the final stage of wound healing.^12^, ^13^ The maturation process mainly includes the degradation of excess collagen fibres by collagenase, rearrangement of collagen and regression of overgrown capillaries, which may last for months to years. Ultimately, the granulation tissue formed in wound healing evolves into normal connective tissue.^13^, ^14^

Numerous endogenous and exogenous adverse factors can disrupt the physiological healing processes, among which the inflammatory phase is the most susceptible to interference. Wound tissue produces various proinflammatory cytokines and chemokines at the initial step in the inflammatory phase, which results in the infiltration of neutrophils and macrophages at injured sites. Neutrophils are required to remove debris and digest invading bacteria through phagocytosis, releasing caustic proteolytic enzymes and producing free radicals in the process of their cleansing activities. Additional cells present in wound sites include macrophages, which mediate angiogenesis, fibroplasia and extracellular matrix (ECM) production, thereby bridging the inflammatory and proliferative phases.^15^ Importantly, moderate inflammation facilitates the removal of necrotic tissue, kills local bacteria and promotes wound healing. However, excessive inflammatory infiltration interferes with normal healing events, such as collagen deposition, angiogenesis and granulation tissue formation. Therefore, it is imperative for inflammation in the wound to be precisely modulated at a level suitable to promote wound healing yet prevented from reaching a level that impedes it.

### 1.2 Hydrogel dressings

Unlike traditional dressings, such as bandages and gauzes, hydrogel dressings are widely acknowledged for their excellent properties, including mechanical properties that are compatible with biological tissues and exceptional water retention capacity which can keep the wound moist and continuously absorb exudate. In addition, their opportune biodegradation avoids secondary damage during dressing replacement, making them ideal wound dressing materials.^16^, ^17^, ^18^, ^19^ Furthermore, compared to other emerging dressings, such as foam and films, hydrogels possess a three‐dimensional porous network structure similar to that of a natural ECM, providing a framework for cells to proliferate and migrate. More importantly, hydrogel dressings can be structurally and biochemically designed and functionally integrated to acquire various advantageous properties,^20^, ^21^, ^22^, ^23^, ^24^ of which anti‐inflammatory hydrogel dressings are foremost representatives. Some commercial hydrogel dressings in wound healing are summarized in Table 1.

**TABLE 1:** Commercial hydrogel dressings in wound healing

**Product name**	**Hydrogel composition**	**Applications**	**References**
Aquaderm	2‐Acrylamido‐2‐methyl‐1‐propanesulfonic acid/2‐hydroxy‐2‐methylpropiophenone/propylene/glycol/polyethylene glycol dimethacrylate	Radiation‐related chronic wounds/mild burns/pressure ulcers	^22^, ^143^, ^144^
INTRASITE Gel	CMC/propylene glycol	Chronic wounds	^22^, ^142^
MEDIHONEY	Glucose oxidase/Leptospermum compounds	Mild burns/surgical incisions/various ulcers	^22^, ^146^
Neoheal Hydrogel	PEG/PVP/Agar	Mild burns/various ulcers/chronic wounds	^22^, ^142^, ^148^
NU‐GEL	SA	Various ulcers	^22^, ^142^
Purilon	CA/SCMC	Various ulcers/mild burns	^22^, ^142^
Restore Hydrogel	HA	Chronic wounds	^22^, ^142^
Simpurity HydroGel	Acrylate/PVA/polyethylene oxide/polyurethane	Mild burns/chronic wounds	^22^, ^142^
SOLOSITE Gel	CMC/glycerol	Various ulcers/mild burns/skin tears	^22^, ^142^, ^149^
Suprasorb G	Acrylic polymers/polyethylene/phenoxyethanol	Chronic wounds/various ulcers/mild burns	^22^, ^142^, ^145^, ^147^

Abbreviations: CA, calcium alginate; CMC, carboxymethyl cellulose; HA, hyaluronic acid; PEG, polyethylene glycol; PVA, polyvinyl alcohol; PVP, polyvinyl pyrrolidone; SA, sodium alginate; SCMC, sodium carboxymethyl cellulose.

**FIGURE 1:**
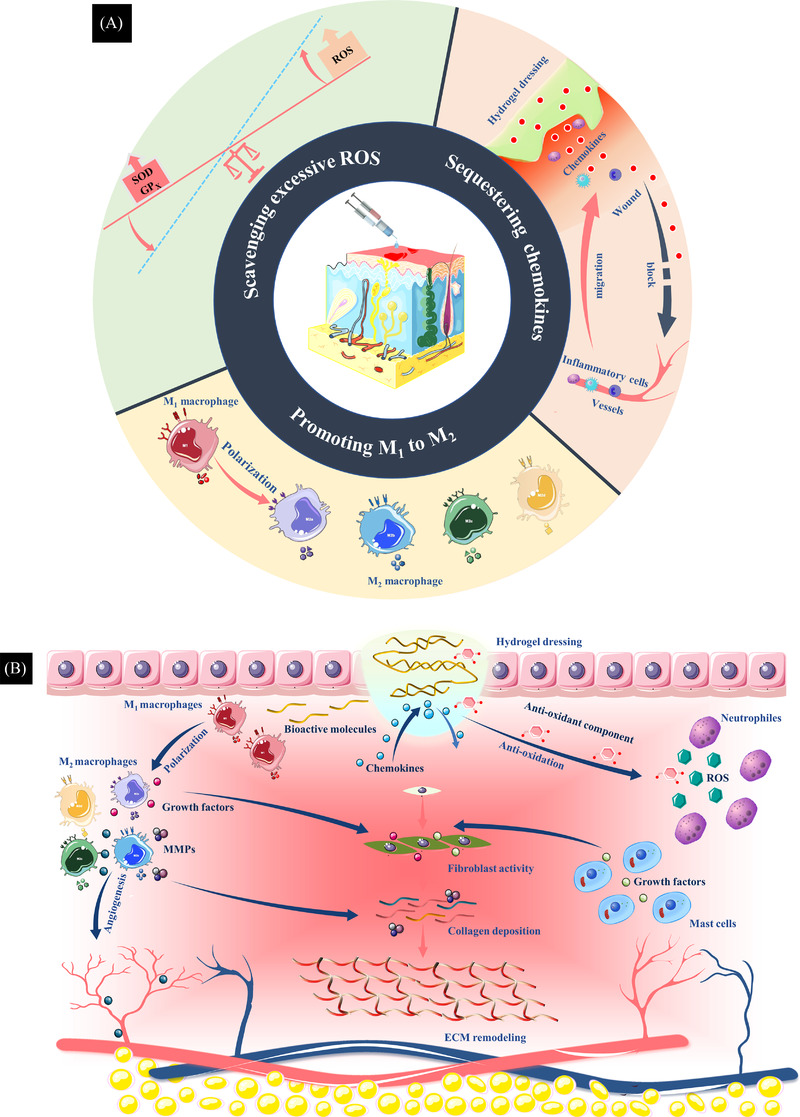
(A) Therapeutic strategies of anti‐inflammatory hydrogel dressings. The categories of anti‐inflammatory hydrogel dressings are based on specific targeted mechanisms of anti‐inflammatory hydrogel dressings: scavenging excessive reactive oxygen species (ROS), sequestering chemokines and promoting M_1_‐to‐M_2_ polarization of macrophages. (B) By integrating drugs, bioactive small molecules and novel biomaterials into the hydrogel matrix, anti‐inflammatory hydrogel dressings can promote angiogenesis, collagen deposition and epithelial cell migration, minimize fibrosis and remodel the extracellular matrix (ECM), promoting wound healing.

In recent years, reactive oxygen species (ROS), chemokines and macrophage phenotypes have been at the centre of research on targeting excessive inflammation in wounds.^20^, ^21^, ^22^, ^25^, ^26^ Natural or synthetic polymers are combined by physical or chemical cross‐linking methods to present different functions and properties. Physical cross‐linking mainly includes hydrophobic association, hydrogen bonding and ionic interactions. The polymers are connected by covalent bonds in chemical cross‐linking, including disulphide, a Schiff base and borate ester bond.^25^, ^26^ The cross‐linking methods depend on the nature of the polymers. Some cross‐linking methods in hydrogel dressings are summarized in Table 2. By integrating drugs, small bioactive molecules and novel biomaterials into a hydrogel matrix, anti‐inflammatory hydrogel dressings can scavenge excessive free radicals, sequester chemokines and promote M_1_‐to‐M_2_ polarization of macrophages, thereby resolving excessive inflammation in the wound and thus promoting wound healing. Over the past 5 years, intensive research has been conducted on anti‐inflammatory hydrogels, but there has been no comprehensive review of anti‐inflammatory hydrogel dressings. Here, we present an overview highlighting the recent achievements in anti‐inflammatory hydrogel dressings, from preparation mechanisms to application methods in wound healing. Categories of anti‐inflammatory hydrogel dressings are based on the specific mechanisms of anti‐inflammatory activities for which hydrogel dressings are created, for example scavenging excessive ROS, sequestering chemokines and promoting M_1_‐to‐M_2_ polarization of macrophages (Figure 1).

**TABLE 2:** Cross‐linking methods in hydrogel dressings

**Cross‐linking methods**	**Interaction modes**	**Hydrogel composition**	**Properties**	**References**
Physical cross‐linking	Hydrophobic association	Hydroxybutyl chitosan/poly(sulfobetaine methacrylate)	Thermosensitive/self‐healing/antibiofouling/antibacterial	^150^
	Hydrogen bonding	Tannic acid/gelatin	Antibacterial/antioxidant/haemostatic/anti‐inflammatory	^151^
	Ionic interactions	Polydopamine/Ag nanoparticles/polypyrrole‐grafted gelatin/ferric ions	Self‐healing/conductive/antibacterial/antioxidant	^152^
Chemical cross‐linking	Disulphide bonds	Keratin/Au(III) salt/deferoxamine	Injectable/biocompatible/haemostatic	^153^
	Schiff base	Quaternized chitosan/benzaldehyde‐terminated Pluronic F127/curcumin	Self‐healing/adhesive/biocompatible/haemostatic/PH responsive/antioxidant	^154^
	Borate ester bonds	Hyaluronate methacrylate grafted with phenylboronic acid/catechin	Glucose responsive/biocompatible/antioxidant	^155^

## 2 SCAVENGING EXCESSIVE ROS

Radical derivatives of O_2_ are known as ROS, well‐known members of which include hydroxyl radicals (•OH), superoxide anion (•O_2_^−^), peroxide (•O_2_^−2^), hydroxyl ions (OH^−^) and peroxide (•O_2_^−2^).^27^, ^28^ Low concentrations of ROS have been demonstrated to facilitate the wound healing.^29^, ^30^ For example, as secondary messengers of inflammatory cells during wound healing, ROS coordinate the recruitment of lymphocytes to a wound, as demonstrated in the ability of a 10 μM concentration of H_2_O_2_ to act as a chemotactic agent for inflammatory cells, a function that is independent of blood‐bound signal components.^27^ In addition, ROS regulate angiogenesis and optimize blood perfusion into the wound‐healing area, and 100 μM concentration of H_2_O_2_ can stimulate angiogenesis via VEGF (vascular endothelial growth factor) signalling.^27^ Furthermore, a burst of ROS induced by phagocytes play a significant role in host defence against invading microorganisms.^31^

However, adverse factors in a wound, such as hyperglycaemia and severe infection, bring a persistent infiltration of inflammatory cells, predominantly neutrophils and macrophages that produce large amounts of ROS with detrimental effects.^32^ In addition, antioxidant capacity is inherently limited in tissues and, when it is relatively deficient, ROS will destroy the structure of DNA, proteins and cell membrane lipids, leading to cell damage and apoptosis.^33^, ^34^ Thus, tissue damage activates a cascade of inflammatory responses, which induces oxidative stress causing persistent inflammatory infiltration, thus initiating a vicious cycle that results in advancing the deterioration of the wound environment.^35^, ^36^, ^37^ Therefore, there is an urgent need for effective strategies to scavenge excessive ROS in wounds.

Recently, various antioxidant components introduced into hydrogels individually or integrated into multifunctional hydrogels through simple combinations, modifications and polymerizations have functioned successfully in scavenging excessive ROS in wounds effectively facilitating wound healing.^38^ Depending on their nature, these components are classified into five categories: natural polyphenols, polysaccharides, amino acids, synthetic polymers^39^ and new metal nanomaterials. The following is an elaboration according to the antioxidant components in the hydrogel dressings summarized in Table 3.

**TABLE 3:** Antioxidant components in hydrogel

**Category**	**Antioxidant component**	**Hydrogel composition**	**Effect of hydrogel dressings**	**References**
Natural polyphenols	Curcumin	Curcumin/quercetin/gelatin	Reduced H_2_O_2_‐induced oxidative stress of cells and proliferation of methicillin‐resistant *Staphylococcus aureus*	^46^
	Resveratrol	Resveratrol/curcumin/alginate	Showed superior antioxidant capability and antibacterial activity and improved cell viability	^49^
	Gallic acid	Gallic acid/gelatin hydroxyphenyl propionic	Scavenged the DPPH radicals and hydroxyl radicals and accelerated the wound healing process	^51^
	Ferulic acid	Feruloyl‐modified peptide/glycol chitosan	Enhanced the regeneration of the epithelium and connective tissue	^53^
	Tannic acid	Tannic acid/PVA/PEG/carboxylated chitosan/HA	Accelerated collagen deposition, decreased TNF‐α levels and facilitated the expression of VEGF	^55^
Polysaccharide	Dextran	Carboxy betaine dextran/sulfobetaine dextran	Showed a faster healing rate than natural dextran hydrogels and a commercial wound dressing (DuoDERM film)	^64^
	Alginate	Oxidized alginate/gelatin/chitooligosaccharide and salicylic acid conjugates	Exhibited improved antioxidant activity and promoted wound healing	^67^
	Paramylon	Paramylon	Resolved wound inflammation and facilitated angiogenesis to promote wound healing	^68^
Amino acids and peptides	Arginine	Arginine derivatives/dopamine‐functionalized HA	Showed greater DPPH, hydroxyl radical scavenging rates and better wound healing outcomes than the HA‐DA hydrogel	^72^
	Silk fibroin peptide	Silk fibroin peptide–grafted hydroxypropyl chitosan/oxidized microcrystalline cellulose/tetramethylpyrazine	Exhibited excellent antioxidant capability and accelerated wound healing process while impeding scar formation	^75^
	Pearl peptides	Pearl peptides/selenium‐containing block‐functionalized PEG/polypropylene glycol	Improved skin fibroblast viability, reduced oxidative stress of cells and promoted angiogenesis in wound healing	^77^
Synthetic polymer materials	Polyvinyl alcohol	PVA/GM‐CSF/mupirocin	Decreased the ROS level and upregulated M_2_ phenotype macrophages in the wound	^78^
	PEA	PAA/PEA	Possessed high hygroscopicity and antioxidant properties, allowing it to absorb and interact with exudates, thereby scavenging ROS	^79^
	Dopamine	Dopamine‐substituted multidomain peptide	Shortened the inflammatory stage of the healing process significantly	^82^
Novel metal nanomaterials	Se nanoparticles	Selenium nanoparticles/bacterial cellulose/gelatin	Showed superior antibacterial activity and outstanding antioxidant capability	^93^
	CeO_2_ nanoparticles	CeO_2_ nanoparticles/chitosan	Showed high antioxidant activities and antibacterial effect, significantly enhancing wound healing	^95^
	Cu_5.4_O nanoparticles	Cu_5.4_O nanoparticles/star‐shaped PEG/heparin	Adsorbed the inflammatory chemokines MCP‐1 and IL‐8, scavenged ROS from exudate to reduce oxidative stress and promoted angiogenesis	^96^

Abbreviations: DPPH, 1′‐diphenyl‐2‐picrylhydrazyl; HA, hyaluronic acid; IL‐8, interleukin‐8; MCP‐1, monocyte chemotactic protein‐1; PAA, polyacrylic acid; PEA, polyesteramide; PEG, polyethylene glycol; PVA, polyvinyl alcohol; ROS, reactive oxygen species; TNF‐α, tumour necrosis factor‐α; VEGF, vascular endothelial growth factor.

### 2.1 Natural polyphenols

**FIGURE 2:**
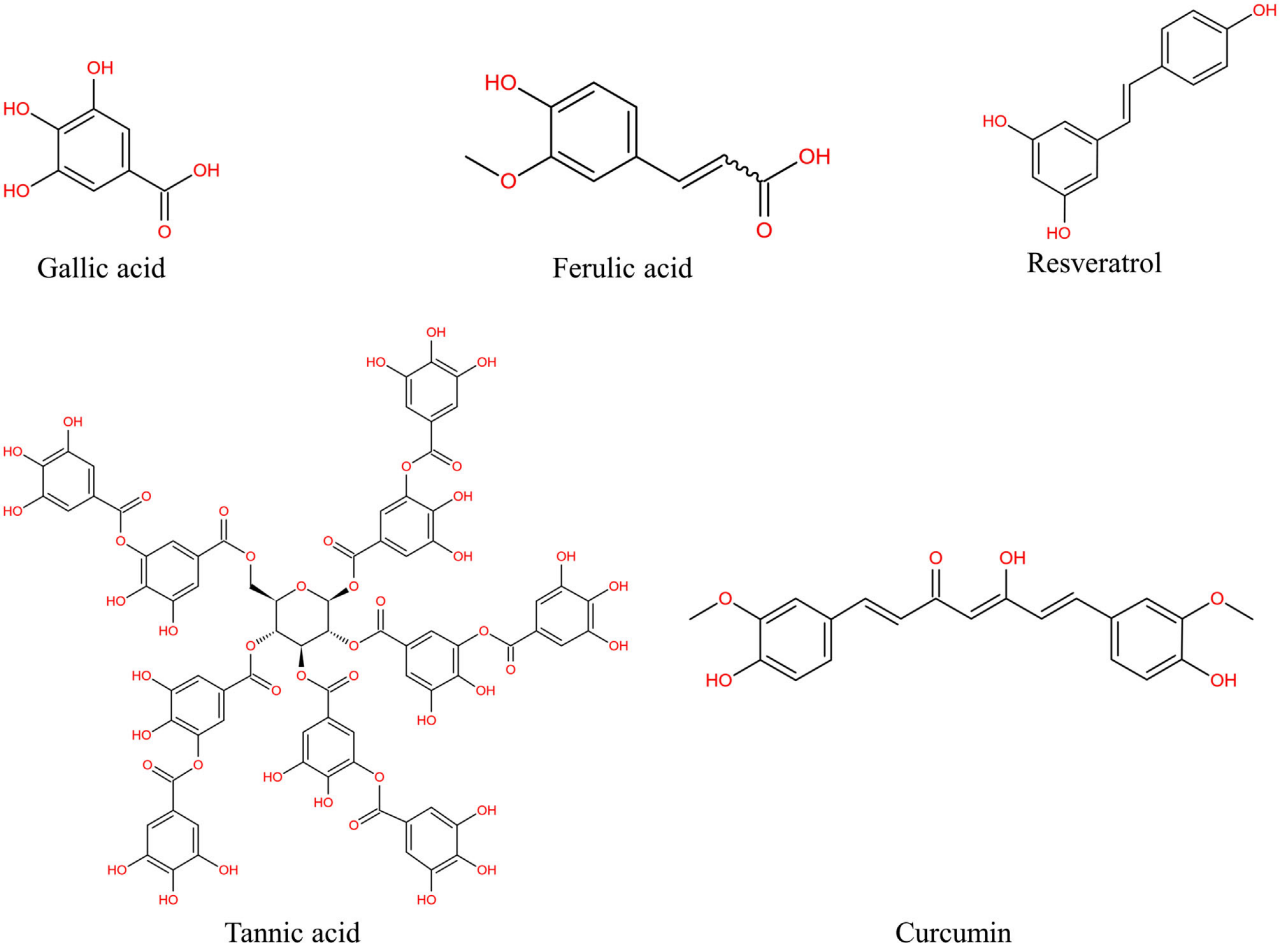
Structure of natural polyphenols

The phenolic hydroxyl groups of natural polyphenols can stabilize ROS through hydrogen shift and electron transfer reactions. In addition, these groups of natural polyphenols chelate transition metals, protect and activate antioxidant enzymes and inhibit oxidative enzymes from resisting oxidative stress.^40^ Furthermore, it is worth noting that some natural polyphenols possess outstanding antimicrobial activity.^41^ Natural polyphenols mainly comprise flavonoids (quercetin, geranin, catechin, catechol, curcumin etc.) and acid ester polyphenols (ferulic acid, gallic acid, tannic acid, derived esters etc.) (Figure 2).^42^, ^43^, ^44^

Curcumin (Cur), the main active ingredient of turmeric, possesses potent anti‐infective, antioxidant and anti‐inflammatory activities,^45^ which is a promising agent for topical use on wounds. di Luca et al. constructed a multifunctional composite prepared by combining hydrogels loaded with curcumin and microparticle systems containing the antimicrobial polyphenol, quercetin.^46^ The final composite hydrogel system was demonstrated to reduce H_2_O_2_‐induced oxidative cell stress and proliferation of methicillin‐resistant *Staphylococcus aureus*. In addition, Yang et al. introduced a thione group into a carboxymethyl chitosan (CS) hydrogel,^47^ and the resulting hydrogel significantly accelerated the wound healing process by inhibiting oxidative cell damage.

Resveratrol, a polyphenolic antioxidant compound, is promising in wound healing for its excellent capacity for modulating tissue regeneration, production of cytokines and insulin sensitivity.^48^ Comotto et al. functionalized an alginate dressing with the natural antioxidants curcumin and resveratrol to enhance its anti‐inflammatory and antibacterial action.^49^ The antioxidant compounds functioned as antibacterial agents and improved cell viability.

Gallic acid, an important phenolic compound, possesses unique properties, including anti‐inflammatory, antimicrobial and free radical scavenging activities.^50^ Thi et al. created an injectable hydrogel by introducing the antioxidant gallic acid–conjugated gelatin into a gelatin–hydroxyphenyl propionic hydrogel.^51^ The antioxidant hydrogel scavenged hydroxyl radicals and 1′‐diphenyl‐2‐picrylhydrazyl (DPPH) radicals, efficiently accelerating the wound healing process.

Ferulic acid, a hydroxycinnamic acid present in the plant cell wall, is a natural antioxidant.^52^ Wei et al. prepared an antioxidant supramolecular hydrogel based on feruloyl‐modified peptides and glycol CS through a mild laccase‐mediated cross‐linking reaction.^53^ The prepared feruloyl antioxidant hydrogel enhanced the regeneration of the epithelium and connective tissue.

Tannic acid, a natural polyphenol derived from plants, has been widely employed in biomaterial design, including surface functionalization, protein modification and cross‐linking of biomaterials.^54^ For example, Li et al. introduced tannic acid into a bilayer hydrogel, forming a dual cross‐linked network and endowing the hydrogel with adhesive properties, antibacterial activity and antioxidant capacity.^55^ The resulting hydrogel significantly accelerated collagen deposition, facilitated the expression of VEGF and decreased tumour necrosis factor‐α (TNF‐α) levels.

In recent years, natural polyphenols have been widely recognized for their biomedical applications. However, the low utilization rate from raw materials and lack of stability in hydrogels limit their development. These aspects still need to be improved for better application in the bioengineering field.

### 2.2 Polysaccharides

Polysaccharides, comprising repeating units of monosaccharides, are widely applied in medical domains, including drug delivery, wound dressing, tissue engineering and bioimaging.^56^ The structure of polysaccharides, rich in hydroxyl and carboxyl groups, forms the basis of hydrogen shift and electron transfer reactions. Polysaccharides exert antioxidant effects through the following two strategies: (1) scavenging free radicals directly or indirectly and (2) increasing the activity of antioxidant enzymes or decreasing the activity of oxidative enzymes.^57^, ^58^, ^59^, ^60^ Meanwhile, polysaccharides can interact with a wide range of biomolecules (nucleic acids, proteins and phospholipids), providing ample scope for the design of hydrogel applications that incorporate their specific beneficial, wound healing attributes.^61^, ^62^

Dextran, a natural polysaccharide produced by bacteria, possesses excellent water retention capacity, acts as a mild scavenger of ROS and reduces platelet hyperactivation.^63^ Qiu et al. constructed a zwitterionic dextran–based hydrogel utilizing carboxybetaine dextran and sulfobetaine dextran.^64^ The prepared hydrogel dressing showed a faster healing rate than both natural dextran hydrogels and a commercial wound dressing (DuoDERM film) due to its excellent antioxidant capacity.

Alginate, extracted from seaweed species, has been widely utilized in biomedical field for their high biocompatibility.^65^, ^66^ Oh et al. fabricated oxidized alginate and gelatin hydrogels loaded with chitooligosaccharide and salicylic acid conjugates synthesized by grafting polymerization.^67^ The resulting hydrogel exhibited improved antioxidant activity and thus accelerated wound healing process. In addition, Lei et al. constructed hydrogels utilizing paramylon derived from *Euglena gracilis* with intrinsic antioxidant and anti‐inflammatory properties.^68^ The prepared hydrogel could effectively resolve wound inflammation and facilitate angiogenesis to promote wound healing.

For their excellent biocompatibility and extensive interactions with biomolecules, polysaccharides are currently combined with various antioxidant components to prepare hydrogels that are more conducive to clinical applications.

### 2.3 Amino acids and peptides

Various amino acids and peptides can react directly with ROS owing to abundant functional groups, such as amino, hydroxyl, carboxyl and sulphur bonds. In particular, the antioxidant effect of amino acids containing phenolic hydroxyl or sulfhydryl groups is more pronounced.^69^ In addition, these amino acid functional groups present the possibility of cross‐linking and grafting modification with hydrogels.

Arginine, an essential amino acid in humans, assumes enormous importance in cell physiology. The carbon–nitrogen double bond in the guanidine group of arginine endows it with antioxidant properties.^70^, ^71^, ^72^ Zhang et al. developed novel hydrogels (HA‐DA/AD) by introducing arginine derivatives (AD) into dopamine‐functionalized hyaluronic acid (HA‐DA).^73^ The HA‐DA/AD hydrogel showed greater DPPH and hydroxyl radical scavenging rates and better wound healing outcomes than simple HA‐DA hydrogels. Although researchers have attempted to integrate amino acids with hydrogel systems, the molecular mechanism of free radical scavenging of amino acids has not been clearly elucidated, which provides a direction for subsequent research.

Silk fibroin peptide, hydrolysate of silk fibroin obtained from the silkworm cocoons, possesses significant antioxidant properties.^74^ Liu et al. prepared an injectable HMSC hydrogel loaded with tetramethylpyrazine based on silk fibroin peptide–grafted hydroxypropyl CS and oxidized microcrystalline cellulose.^75^ The resulting hydrogel exhibited excellent antioxidant capability and accelerated wound healing process while impeding scar formation.

Pearl peptides, extracted from pearl powder, have strong antioxidant and antibacterial properties.^76^ Liu et al. designed pearl peptide–loaded antioxidant hydrogels utilizing selenium‐containing block‐functionalized polyethylene glycol (PEG)/Polypropylene glycol polymers.^77^ The pearl peptide hydrogels improved skin fibroblast viability, reduced oxidative stress of cells and promoted angiogenesis in wound healing.

Most of these natural materials in hydrogels mentioned earlier have good biocompatibility, but their high enzymatic degradability and low physical and chemical stabilities are a major obstacle to widespread biomedical applications. These properties are particularly important concern in natural materials, which may be resolved by synthetic polymer materials.

### 2.4 Synthetic polymer materials

**FIGURE 3:**
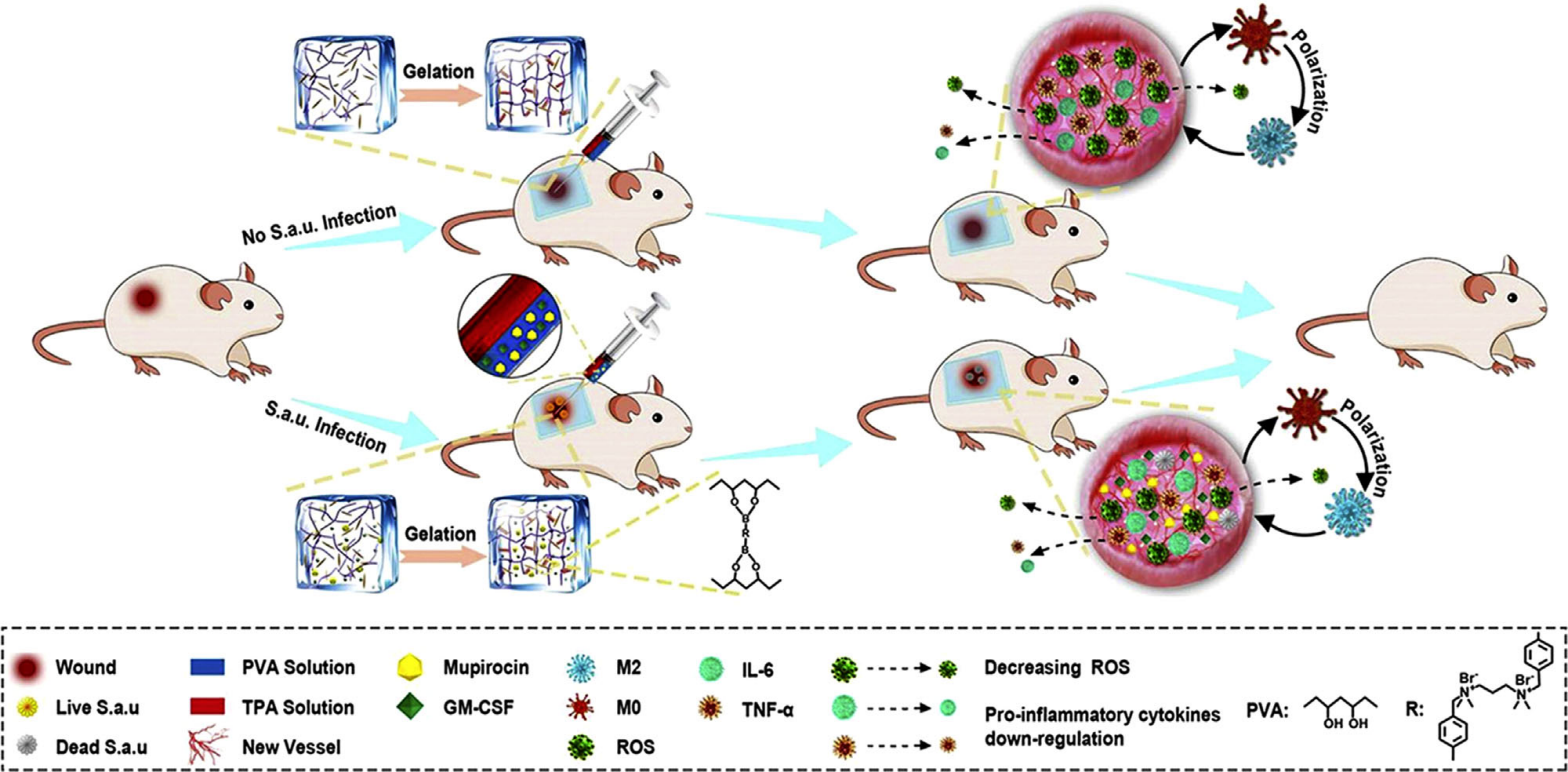
Schematic illustration of the reactive oxygen species (ROS)‐responsive hydrogel loaded with therapeutics for the treatment of bacterially infected wounds.^78^ *Source*: © 2020 Elsevier Ltd.

Over the years, researchers have been inspired to design polymeric materials with excellent properties to compensate for the shortcomings of natural active ingredients.^34^, ^37^ Zhao et al. developed an ROS‐scavenging hydrogel by utilizing polyvinyl alcohol (PVA) cross‐linked by an ROS‐responsive linker.^78^ The resulting hydrogel decreased the ROS level and upregulated M_2_ phenotype macrophages in wounds (Figure 3). Zhang et al. designed a multifunctional hydrogel combining polyacrylic acid (PAA) formed by the cross‐linking polymerization of acrylic acid with an arginine‐based unsaturated polyamide polyesteramide (PEA) through a photopolymerization reaction.^79^ The PAA/PEA hybrid hydrogel possessed high hygroscopicity and antioxidant properties, allowing it to absorb and interact with exudates, thereby scavenging ROS.

Several studies have shown that dopamine‐like substances with abundant catechol groups on their surfaces are considered good scavengers of ROS. The catechol moiety confers antioxidant capacity to dopamine, which scavenges ROS in a dose‐dependent manner.^80^, ^81^ Hussain et al. developed a dopamine‐substituted multidomain peptide with strong skin adhesion, antimicrobial activity and antioxidant capacity.^82^ The dopamine‐substituted multidomain peptide hydrogel significantly shortened the inflammatory phase of the healing process.

Puerarin, a natural plant extract, is characterized by its excellent antioxidant capacity for inhibiting cellular damage and lipid peroxidation. As a complementary therapeutic drug, puerarin is applied with dopamine on wounds to accelerate the healing process.^83^ Herein, Zhang et al. prepared a polydopamine/puerarin nanoparticle–incorporated PEG diacrylate hybrid hydrogel with antioxidant properties.^84^ The prepared hydrogel presented excellent cell proliferation and accelerated regeneration in a whole‐layer skin defect model.

Synthetic polymer materials have overcome some weaknesses of natural active ingredients, but their poor biocompatibility and complex preparation procedures impede their further development. Perhaps an effective combination of natural and synthetic materials would be a better choice, such as PEG and heparin.

### 2.5 Novel metal nanomaterials

Interestingly, it has been discovered that specific metals and their oxides possess antioxidant properties when fabricated into nanomaterials. Selected metal oxide nanoparticles, such as CeO_2_, behave as antioxidant enzymes, that is, nanozymes in pathological conditions, such as superoxide dismutase, catalase and glutathione peroxidase. Nanozymes have substantial catalytically active surface atoms and thus are highly active, whereas natural enzymes usually have only one active centre, thus making them more efficient in catalytic reactions than natural enzymes.^85^, ^86^, ^87^, ^88^

Selenium nanoparticles (SeNPs) are attractive for their prominent anticancer, antiviral and antibacterial activities and significant anti‐inflammatory and antioxidant properties in wound healing.^89^, ^90^, ^91^, ^92^ Mao et al. constructed several multifunctional nanocomposite hydrogel dressings based on bacterial cellulose (BC), gelatin (Gel) and SeNPs.^93^ The decoration of SeNPs endowed the hydrogel with superior antibacterial activity and outstanding antioxidant and anti‐inflammatory properties. In addition, the BC/Gel/SeNPs hydrogel showed excellent performance in skin wound healing.

Nano CeO_2_ has attracted wide attention in nanomedicine due to its extensive applications in drug delivery, biosensing and medicine. Meanwhile, CeO_2_ nanoparticles with outstanding biocompatibility are relatively stable and environmentally friendly.^94^ Ahmed et al. employed a method for the rapid and environmentally friendly synthesis of CeO_2_ nanoparticles from extracts of yellow marshmallow root (*Althaea officinalis*).^95^ A CS hydrogel film, which incorporated the green synthesized cerium oxide nanoparticles, showed high antioxidant activities and antibacterial effects, significantly enhancing wound healing. In addition, Peng et al. developed a composite hydrogel, including Cu_5.4_O ultrasmall nanozymes, for scavenging ROS and star polyethylene glycol (StarPEG) and heparin for sequestering chemokines.^96^ The hydrogel dressing effectively adsorbed the inflammatory chemokines (monocyte chemotactic protein‐1 [MCP‐1] and interleukin‐8 [IL‐8]), suppressing the massive migration of inflammatory cells. In addition, it scavenged ROS from wound exudate, reducing oxidative stress by the sustained release of Cu_5.4_O.

The novel metal and its compound nanomaterials represent a new direction for the development of antioxidant hydrogels. Opportunities that have appeared to date offer a small view towards many materials yet to be discovered for the enhancement of wound healing.

## 3 SEQUESTERING CHEMOKINES

### 3.1 Chemokines

Chemokines, a small family of cytokines, were first identified as substances that aid leukocyte recruitment to sites of injury or infection,^97^ thereby releasing soluble factors to influence the wound healing process. Most chemokine amino acid sequences contain four conserved cysteine residues. According to the polypeptide chain cysteine location, chemokines are classified into four subclasses: C, CC, CXC and CX3C (C indicates cysteine, and X indicates any amino acid), most of which belong to the CC and CXC families. Among them, CXC chemokines are further subdivided according to the presence of glutamate–leucine–arginine (ELR) motifs in front of the first cysteine residue. ELR ^(+)^ chemokines promote angiogenesis, and ELR ^(−)^ chemokines are deemed to possess an angiostatic effect.^98^

**FIGURE 4:**
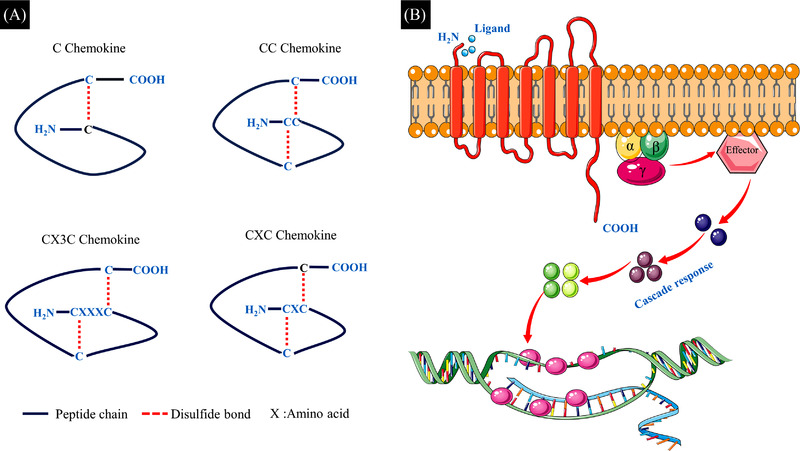
(A) Chemokine family structure. According to the polypeptide chain cysteine location, chemokines are classified into four subclasses: C, CC, CXC and CX3C. The C subfamily is characterized by only one of the proximal N‐terminal cysteines; in the CC subfamily, there are two interconnected cysteines; in the CXC family, the first two cysteines are separated by one amino acid; and in the CX3C family, there are three amino acids between the first two cysteines. The first and third cysteines (C) in the sequence form a disulphide bond, and the second cysteine also forms a covalent bond with the third, stabilizing the tertiary structure of chemokines. (B) Chemokine receptor. They are seven‐transmembrane GPCRs. There are a short acidic N‐terminus outside the cell membrane, binding exclusively to ligand, and three extracellular and three intracellular loops, and the intracellular C‐terminus initiates the intracellular cascade reaction.

Chemokines interact with cells through G protein‐coupled seven‐transmembrane receptors (Figure 4). One type of receptor can bind to multiple chemokines, and the same chemokine can interact with several different types of receptors, allowing chemokines to play important roles in the pathobiological processes of chronic inflammation, tumourigenesis and autoimmune diseases.^99^, ^100^ Involved in all phases of wound healing (haemostasis, inflammation, proliferation, remodelling), chemokines can influence wound healing events, such as angiogenesis, collagen deposition and re‐epithelialization.^101^, ^102^, ^103^ However, persistent chemokine hyperfiltration in wounds has been reported to lead to poor wound healing.^104^, ^105^, ^106^ Therefore, therapeutic strategies targeting excessive proinflammatory chemokines in wounds have been constantly upgraded, and potential approaches to target chemokines include monoclonal antibodies, small‐molecule antagonists and glycosaminoglycans (GAGs) that interfere with the distribution of chemokines.^101^ GAG‐based anti‐inflammatory hydrogels are among the most prominent wound‐healing therapeutic strategies. Hydrogels are prepared by flexibly utilizing various biomimetic materials based on the principle of interaction between GAGs and chemokines to capture excessive proinflammatory chemokines from wounds to promote wound healing.

### 3.2 Glycosaminoglycan hydrogel dressings in wound healing

GAGs, a family of negatively charged linear polysaccharides, are widely found on the surface of human cells and in the ECM.^107^ Capable of interacting with various proteins, including cytokines, growth factors, proteases and chemokines, GAGs mediate physiological processes, such as cell adhesion and intracellular signal transduction. In addition, GAGs participate in multiple diseases, including cardiovascular diseases, neurodegenerative diseases and tumours, through the electrostatic interactions between positively charged amino acid residues and negatively charged sulphate groups.^108^

In complex wounds (such as diabetic and burn wounds), persistent and excessive inflammatory cells infiltration produces massive amounts of proinflammatory chemokines, such as MCP‐1 and IL‐8, which further aggravate the invasion of inflammatory cells into the wound bed, thereby perpetuating chronic inflammation.^109,^ ^110^ One important feature of chemokines is their ability to bind to ECM GAGs, a process mediated by the electrostatic interaction described earlier.^109^ It has been demonstrated that the significant GAG‐binding motif on chemokines is usually BBXB or BBBXXBX, where B and X represent basic and any amino acid, respectively. In addition, specific chemokine‐binding epitopes on GAG have been identified, such as the 2‐*O*‐sulphate group on the allulose unit.^107^ Accordingly, the sulphation pattern of GAGs governs multiple binding events, such as the distribution of chemokines within the ECM, controlling immune cells activation and migration.^110^, ^111^, ^112^

**FIGURE 5:**
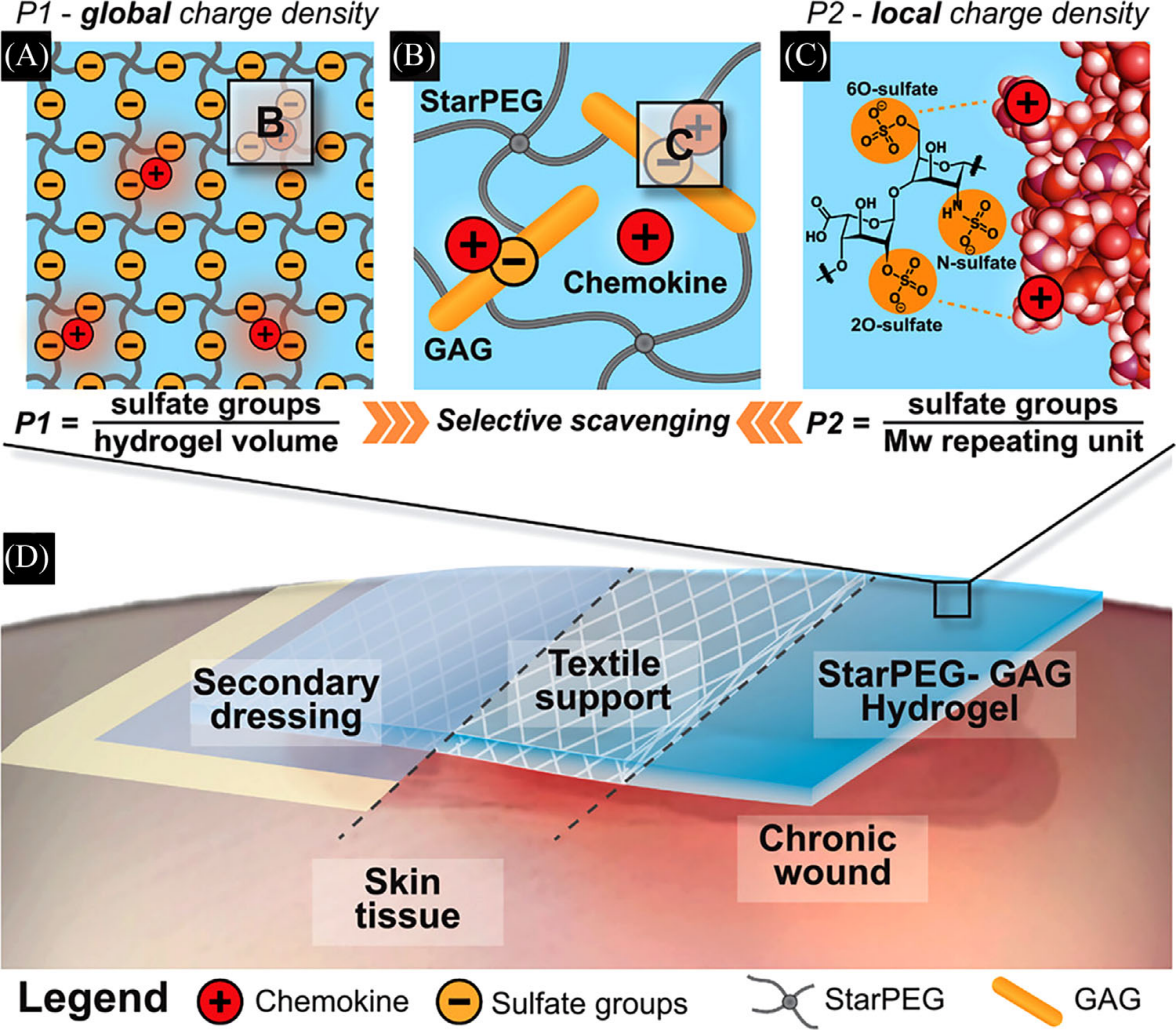
(A) P1 reflects global charge density of glycosaminoglycan (GAG); (B) positively charged patches at the chemokine surface; (C) P2 reflects the local charge density between GAG and single positively charged amino acid residues of the protein surface; (D) a wound contact layer composite dressing is applied on the surface of the chronic wound performing its function.^111^ *Source*: © 2021 The Authors. Advanced Science published by Wiley‐VCH GmbH

Lohmann et al. customized a modular hydrogel based on StarPEG and GAG heparin derivatives to achieve maximal chemokine sequestration.^110^ As a result, the inflammatory chemokines IL‐8, macrophage inflammatory protein 1 and MCP‐1 in wound fluids were effectively scavenged, inhibiting the migration of human monocytes and neutrophils. In addition, the resulting hydrogel showed better performance than the standard‐of‐care product PROMOGRAN with respect to granulation tissue formation, angiogenesis and wound closure in a delayed wound healing model. Similarly, Schirmer et al. developed a wound contact layer based on a StarPEG‐GAG hydrogel.^111^ The composite wound contact layer dampened excessive inflammatory signals without affecting the levels of pro‐regenerative growth factors, promoting wound healing by increasing granulation tissue formation, vascularization and deposition of collagen fibres (Figure 5).

Additionally, Qin et al. prepared a hydrogel that mimics the ECM, consisting of an HA‐derived component with anti‐inflammatory activity and a gelatin‐derived component with adhesion sites for cell anchoring.^112^ The results showed that the HA‐Gel hydrogel was a viable therapeutic option for full‐thickness wounds by effectively depleting the proinflammatory chemokine MCP‐1 in the wound bed.

However, the function of chemokines in wounds is extraordinarily complex. The anti‐inflammatory hydrogel is not intended to sequester or remove all chemokines but to target and sequester chemokines that are detrimental, such as MCP‐1 and IL‐8, thereby promoting wound healing. Conversely, loading special chemokines that facilitate wound healing into hydrogel dressings can accelerate the wound healing process. Xu et al. customized a biomimetic hydrogel utilizing PVA and CS as hybrid materials while loaded with chemotactic factor (SDF‐1) to stimulate a rapid in situ recruitment of bone marrow mesenchymal stem cells (BMSCs) for rapid wound repair and regeneration.^113^ The doped chemokines can be consistently released from the hydrogel and significantly recruit BMSCs in vitro and in vivo. The hydrogel‐based biomimetic PVA/CS hybrids for the local release of chemokines are a promising vehicle to improve healing outcomes without causing scar formation or any other adverse complications.

In fact, based on electrostatic interactions, negatively charged polysaccharides will inevitably deplete some positively charged soluble proteins that facilitate wound healing, such as VEGF. How to dampen this side effect is an urgent issue that needs to be addressed. The sulphation degree and concentration of GAGs in hydrogels maybe a possible research direction.

## 4 PROMOTING M_1_‐TO‐M_2_ POLARIZATION OF MACROPHAGES

### 4.1 Macrophages

Macrophages play a vital role in recognizing and removing pathogens, cellular debris and phagocytosis of apoptotic neutrophils in the early stages of wound healing and enhancing angiogenesis, collagen deposition and epithelial cell migration in the later stages.^114^ Macrophages in the skin are classified into two categories depending on their origin: (1) resident macrophage populations formed before birth and (2) monocytes recruited to the injured area from the circulatory system and then differentiating into macrophages. The first group of macrophages consists of self‐renewing cells produced by the embryonic yolk sac.^115^ Monocyte‐derived macrophages initially migrate to injured areas via damage‐associated molecular pattern or pathogen‐associated molecular pattern signalling. In addition, monocytes are also recruited through damaged blood vessels.^115^, ^116^ Depending on the role of macrophages in wound healing, they are classified into proinflammatory macrophages (M_1_) and anti‐inflammatory macrophages (M_2_). This classification is still controversial, as the origin of anti‐inflammatory macrophages (M_2_), the method of phenotypic transition from M_1_ to M_2_ and the relative proportions of each in macrophage populations all remain obscure. However, it is important to analyse the functions of different types of macrophages in wound healing. Proinflammatory macrophages (M_1_) produce ROS, nitric oxide, IL‐6, TNF‐α, matrix metalloproteinase 9 and so on to recognize and remove pathogens, cellular debris and apoptotic neutrophils in the early stages. Anti‐inflammatory macrophages (M_2_) exert their effects in the proliferative and remodelling phases of wound healing. They bring high levels of growth factors (platelet‐derived growth factor, insulin‐like growth factor 1 etc.), metalloproteinase inhibitor 1, arginase 1 (Arg‐1) and so on to promote angiogenesis, collagen deposition and epithelial cell migration and to minimize fibrosis and remodel the ECM.^116^, ^117^, ^118^, ^119^ Similar to the overlap of stages in wound healing, some macrophages share proinflammatory and anti‐inflammatory phenotypes and may even exert other effects as well.

### 4.2 Polarization of macrophages in wound healing

The polarization of macrophages is highly dependent on the wound microenvironment,^114^ which is dynamic during the healing process, thus affecting the phenotype and functions of macrophages. Adverse factors, such as hyperglycaemia and bacterial infections, impede the polarization of proinflammatory macrophages (M_1_) to anti‐inflammatory macrophages (M_2_).^120^ The wound then remains in the inflammatory phase, impairing epithelial regeneration, collagen deposition and angiogenesis and hindering the wound from shifting to the repair phase. Therefore, how to promote the polarization of the persistent proinflammatory macrophages (M_1_) in wounds becomes a critically urgent problem.

In recent years, various dressings have been designed to regulate the microenvironment of chronic wounds with an expectation of promoting macrophage polarization in the later stages of wound healing. Among them, hydrogel dressings have attracted the greatest attention. Hydrogels can be designed to immunomodulate chronic wounds by delivering bioactive molecules, including antimicrobial molecules, immunomodulatory components, growth factors, genes and cells, promoting the polarization of M_1_ macrophages to M_2_, thus accelerating the wound healing process.^121^ Selected bioactive molecules for use in hydrogel dressings are summarized in Table 4.

**TABLE 4:** Bioactive molecules that promote macrophage polarization

**Bioactive molecules**	**Hydrogel composition**	**Effect of hydrogel dressings**	**References**
PGE_2_	PGE_2_/Chitosan	Modulated the balance between three overlapping phases of inflammation, regeneration and remodelling during wound healing	^124^
Lactic acid	*Lactococcus*/heparin‐poloxamer	Induced M_2_ phenotypic transformation of macrophages and produced and protected VEGF	^126^
Collagen	Modified collagen	Shifted macrophages towards an anti‐inflammatory phenotype and attenuated inflammatory responses	^127^
Paeoniflorin	Paeoniflorin/HA	Promoted the polarization of macrophages from M_1_ to M_2_ and improved angiogenesis, re‐epithelialization and collagen deposition	^132^
H_2_S	JK1(H_2_S donor)/HA	Facilitated the polarization of proinflammatory macrophages (M_1_) to anti‐inflammatory macrophages (M_2_)	^136^
Bioactive glass	BG/SA	Induced the polarization of M_2_ phenotype and enhanced the synthesis of fibroblast ECM and vascularization of endothelial cells	^139^
miR‐223*	miR‐223*/HA	Increased the expression of the anti‐inflammatory gene Arg‐1 and decreased the expression of proinflammatory markers, including TNF‐α, IL‐1β and IL‐6	^140^

Abbreviations: ECM, extracellular matrix; HA, hyaluronic acid; PGE_2_, prostaglandin E_2_; SA, sodium alginate; TNF‐α, tumour necrosis factor‐α; VEGF, vascular endothelial growth factor.

Prostaglandin E_2_ (PGE_2_), secreted by mesenchymal stem cells, can promote the polarization of M_1_ macrophages to alleviate inflammation and accelerate the skin wound healing process.^122^, ^123^ Zhang et al. incorporated PGE_2_ into a CS hydrogel (CS + PGE_2_ hydrogel).^124^ The experimental results showed that the controlled release of PGE_2_ attenuated the inflammatory response by inducing the polarization of M_1_ macrophages to M_2_, and the CS + PGE_2_ hydrogel could modulate the balance between the three overlapping phases of inflammation, regeneration and remodelling in wound healing.

**FIGURE 6:**
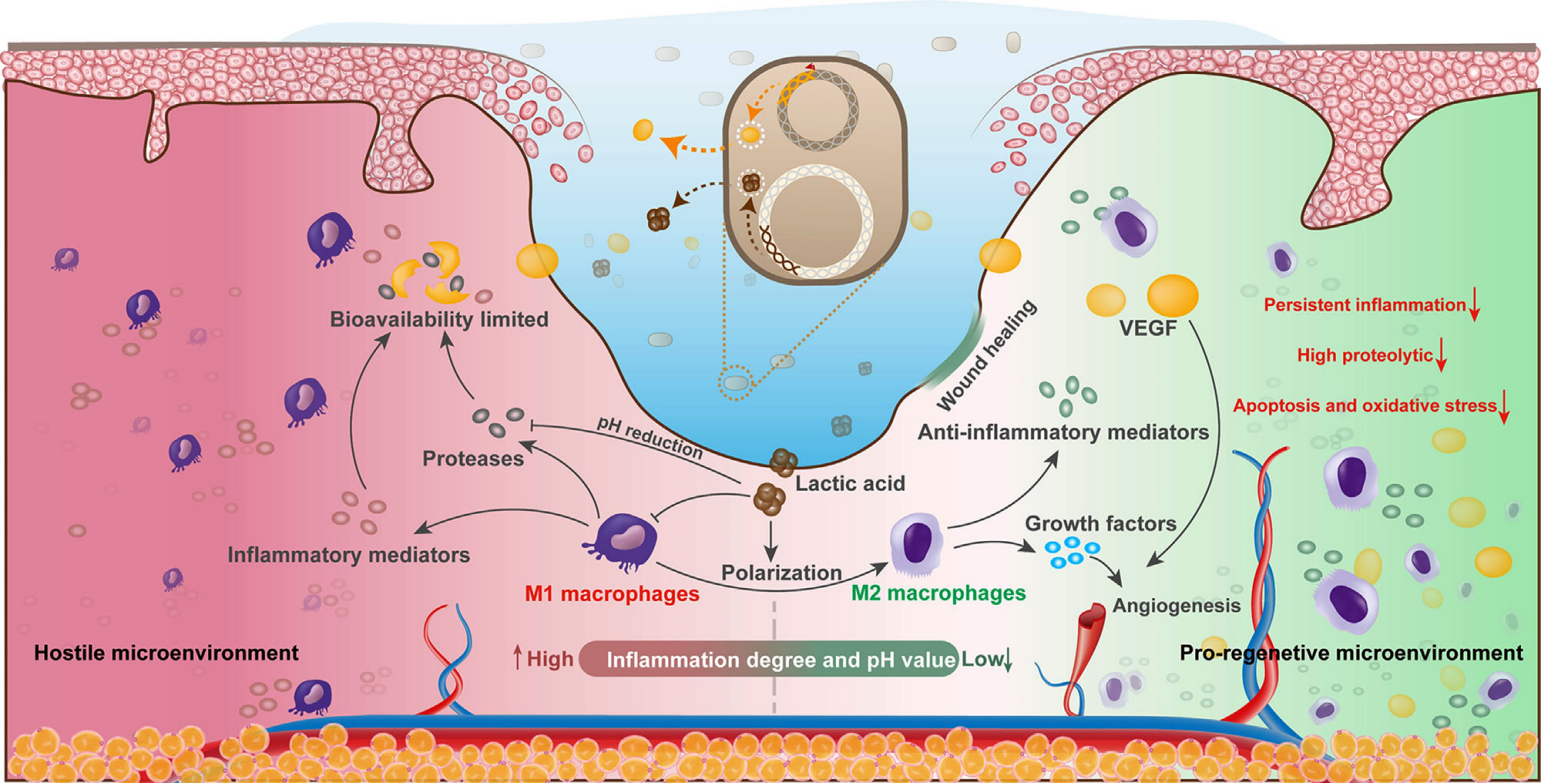
Illustration of the HP@LL_VEGF hydrogel modulating the wound microenvironment to accelerate angiogenesis and wound regeneration.^126^ *Source*: © 2021 The Authors. Advanced Functional Materials published by Wiley‐VCH GmbH

Lactic acid–producing bacteria, the most commonly utilized probiotics, have great effects on protecting the host against microorganisms harmful to the human body, strengthening the host immune system and reducing metabolic disorders.^125^ Lu et al. designed a heparin–poloxamer thermoresponsive hydrogel incorporating a delivery system comprising living *Lactococcus*.^126^ The lactic acid secreted by the living, probiotic system can induce M_2_ phenotypic transformation of macrophages, significantly promoting angiogenesis in diabetic wounds, and the resulting hydrogel can also produce and protect VEGF, increasing proliferation, migration and tube formation of endothelial cells (Figure 6).

Modifying the original dressing via physical or chemical approaches may enhance the functions of hydrogel applications. For example, Das et al. utilized a modified collagen gel dressing, shifting macrophages towards an anti‐inflammatory phenotype and attenuating inflammatory responses.^127^ The resulting hydrogel can change wound inflammation outcomes by increasing the production of anti‐inflammatory IL‐10, IL‐4 and proangiogenic VEGF.

Meanwhile, paeoniflorin, the main bioactive component of the total glucoside extracted from peony, can control macrophage activity.^128^, ^129^, ^130^, ^131^ Yang et al. prepared a high molecular weight HA‐based hydrogel loaded with paeoniflorin extracted from *Paeonia lactiflora*.^132^ The constructed hydrogel dressing significantly promoted the polarization of macrophages from M_1_ to M_2_, and this result was accompanied by improved inflammation, angiogenesis, re‐epithelialization and collagen deposition.

As a small molecule signal transduction motif, H_2_S exerts a therapeutic effect by penetrating the cell membrane, and one of the mechanisms of its therapeutic effect is to promote the phenotypic transformation of macrophages to M_2_ macrophages.^133^, ^134^, ^135^ Herein, Wu et al. incorporated a pH‐controllable H_2_S donor (JK1) into an HA‐based biomimetic hydrogel, thereby constructing a hybrid system.^136^ The resulting hybrid hydrogel can facilitate the polarization of proinflammatory macrophages (M_1_) to anti‐inflammatory macrophages (M_2_), significantly accelerating the wound regeneration process through increased re‐epithelialization, vascularization and deposition of collagen fibres on dermal wounds.

Bioactive glass, a group of materials consisting of SiO_2_, CaO, Na_2_O and P_2_O_5_, is a remarkable bioactive material widely applied in clinical practice for tissue transplantation.^137^ The ion products of bioactive glass have been demonstrated to activate the M_2_ phenotype of macrophages and stimulate the secretion of more anti‐inflammatory growth factors by macrophages.^138^ Zhu et al. explored the function of the bioactive glass/sodium alginate hydrogel and showed that it could induce the polarization of the M_2_ phenotype and upregulate the expression of anti‐inflammatory genes.^139^ In addition, M_2_‐polarized macrophages further recruited fibroblasts and endothelial cells, enhancing the synthesis of fibroblast ECM and vascularization of endothelial cells.

Gene delivery is considered a versatile alternative strategy, of which the main aim is to immunomodulate the phenotype of macrophages, increase anti‐inflammatory cytokine expression, reduce the secretion of proinflammatory cytokines and enhance the recruitment of Treg cells to suppress inflammation. Here, Saleh et al. developed adhesive hydrogels containing miR‐223 5p mimic (miR‐223*) to control the polarization of macrophages in wounds.^140^ These hydrogels demonstrated the upregulation of miR‐223* in J774A.1 macrophages, increased the expression of the anti‐inflammatory gene Arg‐1 and decreased the production of proinflammatory markers, including TNF‐α, IL‐1β and IL‐6.

The level of inflammation in wounds is dynamic, and the phenotype of macrophages varies according to the wound microenvironment. Macrophages display different phenotypes to perform various roles during the wound healing process. They exhibit a proinflammatory M_1_ phenotype in the early inflammatory stages and an anti‐inflammatory M_2_ phenotype in the repair stages. A phenotypic continuum may exist during the process, with some cells sharing the phenotypic characteristics of M_1_ and M_2_ macrophages.^141^ The phenotypic regulation of macrophages is a sophisticated process. Insufficient M_1_ macrophages in the early stages may lead to severe infection or delayed wound healing, whereas excessive M_2_ macrophages in the later stages may result in scar formation. However, current dressings lack the ability to precisely modulating the phenotype of macrophages to achieve predictably ideal results. In addition, few studies on hydrogels have uncovered the molecular mechanisms of macrophage polarization, which is of great significance for the precise regulation of macrophage activity in wound healing. Therefore, more research is needed to solve this major issue.

## 5 CONCLUSION AND PERSPECTIVES

The regulation of inflammation in wounds is complex. Insufficient inflammation levels in the early stages and excessive inflammation infiltration in later stages both lead to the disruption of the healing process, with the latter being more common in wound healing. Therefore, various advanced anti‐inflammatory biomaterials have been used in wound healing in recent years, especially in treating chronic wounds. Among them, anti‐inflammatory hydrogel dressings can chemically, mechanically and electrically mimic skin functions and, thus, have attracted considerable attention. This review summarizes the scope of emerging anti‐inflammatory hydrogel dressings, focusing on three required aspects of wound healing: scavenging excessive free radicals, sequestering chemokines and promoting M_1_‐to‐M_2_ polarization of macrophages. However, the development of anti‐inflammatory hydrogel dressings for enhanced wound healing is still in the early stages. These approaches also face unique challenges related to their biocompatibility, technology and clinical results, which must be addressed before the previous treatments can be transformed into clinical applications. Unfortunately, animal models for which treatments have been evaluated cannot fully replicate the complexity of chronic wound healing in humans, and human physiology is vastly distinct from that of the mouse. In addition, the safety of hydrogels must be evaluated carefully, as they may trigger inappropriate immune responses, such as infections, allergies and autoimmune diseases. Therefore, intensive research is needed to allow the creation of promising anti‐inflammatory hydrogel dressings for successful use in clinical applications.

In the future, anti‐inflammatory hydrogel dressings may afford opportunities for a precise modulation of wound healing processes, with real‐time monitoring of wound inflammation levels using wearable sensors and imaging devices, perhaps with automated stimulus responsiveness and other technologies to adjust therapeutic strategies. Such advances in the development of precise treatment of wounds will improve patient curing rates, alleviate pain and reduce costs. Meanwhile, anti‐inflammatory hydrogels may also be loaded with other functional components, such as haemostatic, conductive and adhesive materials, making anti‐inflammatory hydrogel dressings more powerful and predictable for clinical applications, which is a vitally important subject in translational research, particularly in the use of advanced anti‐inflammatory hydrogel dressings. Translational research may generate clinically meaningful outcomes in wound healing that improve human health and allow fundamental scientific findings to be translated more efficiently into practical applications. Clearly, this will require the concerted efforts of a wide range of researchers and clinicians, but the outcomes can be transformative.

## CONFLICT OF INTEREST

The authors declare no conflict of interest.
